# The Practice Effect on Time-Based Prospective Memory: The Influences of Ongoing Task Difficulty and Delay

**DOI:** 10.3389/fpsyg.2019.02002

**Published:** 2019-08-28

**Authors:** Yunfei Guo, Peiduo Liu, Xiting Huang

**Affiliations:** Key Laboratory of Cognition and Personality, Ministry of Education, Faculty of Psychology, Southwest University, Chongqing, China

**Keywords:** time-based, prospective memory, practice effect, task difficulty, delay

## Abstract

The practice effect on prospective memory refers to the phenomenon that prospective memory performance can improve with behavior training. Some studies have found that event-based prospective memory (EBPM) can benefit from practice. However, only a few studies have focused on the practice effect on time-based prospective memory (TBPM). In the present study, we planned to explore whether the practice effect on TBPM existed and what its processing mechanism was. In Experiment 1, we tested whether the practice effect existed at all under different background task conditions. The results showed that the practice effect existed only under an easy ongoing task condition. When a 600 ms delay was added after each difficult ongoing task in Experiment 2, we found the same effect as for the easy ongoing task condition in Experiment 1. In addition, the results also suggested that the practice effect was closely related to the improvement in the effectiveness of time monitoring. The present study confirmed the existence of practice effect of TBPM under some conditions of sufficient attention resources and further explored its causes for the first time, which made us have a deeper understanding of the plasticity of TBPM caused by behavior training.

## Introduction

Prospective memory refers to the ability to remember to carry out an intended action at the appropriate time or during an appropriate situation in the future (Einstein and McDaniel, 1990). According to the nature of cues, prospective memory can be divided into event-based prospective memory (EBPM) and time-based prospective memory (TBPM). EBPM needs to be executed when specific situations or clues appear. TBPM needs to be executed at a definite time point or after a certain time span (Einstein and McDaniel, 1990; Han et al., 2017). Prospective memory consists of prospective component referring to remembering that something has to be done and retrospective component referring to what has to be done (Meier and Zimmermann, 2015). Prospective memory has serious impacts on people’s lives. Many studies focus on how to improve prospective memory performance through behavior training (Waldum et al., 2016). However, some studies further divided behavior training into strategy training and cognitive process training (Brom and Kliegel, 2014; Hering et al., 2014). Strategy training mainly trained individuals to use efficient coding strategy, while cognitive process training focused on how to improve the cognitive ability related to prospective memory. We focus on cognitive process training.

Repeated training for specific prospective memory task can also effectively improve the prospective memory performance (Yip and Man, 2013; Blondelle et al., 2016). This phenomenon is defined as the practice effect on prospective memory in the present study. Does the practice effect exist in TBPM? To date, several studies focused on the practice effect on TBPM. Two of them found that the performance of regular TBPM tasks (which appeared more than five times) was better than that of irregular TBPM tasks (which only appeared once) in both younger and the older adults (Hu and Feng, 2013; Blondelle et al., 2016). Another study found that the activation of cerebral cortex associated with TBPM decreased and showed plasticity after repeated training for TBPM tasks (Rose et al., 2015). However, the TBPM tasks mentioned in the above studies were displayed in text on a computer screen. Their successful executions depended not on accurate time estimation and adequate time monitoring, but on the monitoring of written clues. These prospective memory tasks were not the real TBPM tasks, but the EBPM tasks. However, Waldum et al. (2016) created 8 training programs (including coding strategy training and repetitive prospective memory training) to improve the prospective memory ability of older adults. The results showed that the TBPM performance in the training condition was better than that in the control condition after training. Currently, evidence supporting the existence of the practice effect on TBPM is limited. In theory, however, TBPM is likely to benefit from practice. The dynamic attending theory (DAT) has proven to be a reasonable explanation for TBPM (Jones, 2006). The DAT points out that when processing time information, we would allocate attention resources according to the properties of time stimulations. Under the circumstance of regular stimuli (high frequency and a fixed time interval), our attention will develop a “narrow focus” (allocating more attention to the time point of the target) due to good time expectations (Large and Jones, 1999; Herrmann and Henry, 2014; Qiu et al., 2017). According to the DAT, training for a specific TBPM task can help individuals form good time expectations, leading to improvement the performance of TBPM. The above evidence has indicated that a practice effect might exist for TBPM.

Some studies have found that the practice effect on EBPM was affected by task difficulty. At least, when the ongoing task was difficult, a small amount of exercises could hardly promote the performance of prospective memory (McDaniel and Scullin, 2010). The ongoing task difficulty mainly indirectly affected the amount of attention resources allocated to prospective memory tasks. The attention resource theory argued that multiple non-automated tasks together occupied a limited amount of attention resources together (Kahneman, 1973). In the dual-task paradigm, prospective memory tasks and ongoing tasks compete for limited attention resources. Compared with easy ongoing task, the difficult ongoing tasks occupy more attention resources, which could lead to less attention allocation to prospective memory (Meier and Zimmermann, 2015; Guo et al., 2016). However, the processing of temporal information must require a certain amount of attention resources (Cruz et al., 2017). Under the condition of a high-difficulty ongoing task, participants could not devote enough attention to temporal information processing at least, leading to poor accuracy of time estimation (Coyne et al., 2009). Under the insufficient attention condition, the deviation in time estimation was also greater than that under the sufficient attention condition even after training (Taatgen et al., 2007), and the time expectations were still poor. However, a low-difficulty ongoing task did not significantly interfere with a prospective memory task. Therefore, the practice effect on TBPM is likely to be constrained by a difficult ongoing task.

If the practice effect on TBPM was not found under the difficult ongoing task condition, could we take certain measures to make the practice work? It is generally known that a difficult ongoing task occupies most of the attention resources in the dual-task paradigm and the prospective memory task is disturbed by insufficient attention. This disruption was probably the main reason that the practice effect was not been found in the difficult background task condition. The delay theory held the view that when the ongoing task led to overload, prolonging the presentation time of the task stimulus could provide additional attention resources for the processing of the prospective memory task, improving the performance of prospective memory task (Mcbride et al., 2013; Heathcote et al., 2015; Strickland et al., 2017). Loft and Remington (2013) found that adding a delay of more than 200 ms after each ongoing task could significantly improve prospective memory performance. According to the delay theory and the evidences above, providing an additional delay could provide enough attention resources and more time for rehearsal. Thus, the delay was likely to eliminate the interference of the task difficulty with the practice effect. The second goal of the present study was to validate the prediction of the delay theory.

If the practice effect exists for TBPM, how does it come into being? We have speculated that the monitoring times would increase near the time point of the task execution in the paragraph 4. Some studies found that there was a positive correlation between the number of time monitoring near the time point of task execution and the performance of TBPM (Yuan et al., 2011; Doyle et al., 2013; Mioni and Stablum, 2014), which confirmed the speculation. Therefore, the closer the monitoring behavior was to the time point of task execution, the more effective the time monitoring was. Beyond that, some studies found that the increase in monitoring times could also significantly improve TBPM performance (Mioni and Stablum, 2014; Vanneste et al., 2016). Therefore, the increase in the total attention input into temporal information processing may be a possible cause for the practice effect. In addition, the difference between TBPM and EBPM lies in the prospective component. The TBPM task cannot reach the level of spontaneous processing, because the temporal information processing of the prospective component must cost attention resources. However, the retrospective component of TBPM involves only retrospective memory. It reduces the dependence on attention resources after practice, resulting in the better performance of TBPM tasks (Coane, 2013; Boywitt et al., 2015). Therefore, the improvement of the spontaneous processing of the retrospective component might be one of the reasons for the practice effect.

The present study had two major aims. The first aim was to systematically explore whether the practice effect on TBPM exists under different ongoing task conditions. Our second goal was to explore the cognitive processing mechanism of the practice effect. We planned to use two experiments to answer the questions. In Experiment 1, we investigated whether the practice effect was affected by the difficulty of the background task, and preliminarily tested the cognitive processing mechanism through multiple indicators. We speculated that the practice effect existed only under the easy ongoing task condition. If we did not find the practice effect under difficult ongoing task condition, the reason might be that prospective memory task did not get enough attention resources. Adding a delay could provide more time to process TBPM task, which would be conducive to the generation of the practice effect. Therefore, in Experiment 2, we created a slow-pace difficult ongoing task to examine whether the practice effect existed under a difficult background task condition when participants were provided with sufficient time for TBPM through adding a 600 ms delay. In addition, we also compared the differences of multiple indicators between Experiment 1 and Experiment 2 under difficult ongoing task condition, which would further validate the processing mechanism of the practice effect on TBPM.

## Experiment 1

There were two purposes of Experiment 1. The first purpose was to explore whether the practice effect on TBPM existed under different cognitive loads of ongoing task. We planned to use the n-back (*n* = 1, 2) paradigm to manipulate the task difficulty of the ongoing task. The 2-back task was a difficult and constantly updated working memory task. With inadequate practice, its performance was not easy to improve, which might interfere with the effect of practice in improving TBPM task performance. Therefore, we assumed that TBPM tasks would benefit from short-term practice under the 1-back task condition, but not under the 2-back condition. The second aim was to test the cognitive processing mechanism of the practice effect. We would pay attention to whether the relevant indicators changed accordingly when the practice effect emerged.

### Method

#### Participants and Design

In previous studies related to practice effect of TBPM, the number of participants at each level ranged from 15 to 36 (Hu and Feng, 2013; Rose et al., 2015; Blondelle et al., 2016; Waldum et al., 2016). But a larger number of participants could reduce the influence of random errors and made the experimental results more reliable (Drazen et al., 2016). In view of the above two points, more than 30 participants were adopted at each level of the two experiments in the present study.

One hundred and forty-eight university students participated in Experiment 1 in exchange for monetary compensation (30 RMB, about 4.5 dollars). They were tested individually. Their ages were between 18 and 24 years (*M* = 20.61, *SD* = 1.44). The experiment adopted a 2 (training conditions: control condition/experimental condition) × 2 (task difficulty: easy task/difficult task) between-subject design. In the control condition, participants practiced the ongoing task only in the training stage, but the other participants needed to practice both the ongoing task and the prospective memory task in the experimental condition. They were randomly assigned to the conditions of control condition/easy task, experimental condition/easy task, control condition/difficult task, and experimental condition/difficult task, respectively. According to the criteria mentioned in the results, we obtained thirty-three, thirty-four, thirty-two, and thirty effective participants in the conditions of control condition/easy task, experimental condition/easy task, control condition/difficult task, and experimental condition/difficult task, respectively. The research was approved by the Academic Committee of Southwest University. Before participating in the experiment, participants were informed about the general task content and the matters needing attention in advance through the network communication software. They could decide whether to take part in the experiment according to their own wishes.

#### Materials, Tasks, and Apparatus

The materials for the ongoing tasks were 26 English capital letters. The ongoing tasks were the *n*-back (*n* = 1, 2) tasks. The 1-back task required participants to compare the current letter with the first letter preceding it. If the two letters were the same, the participants were instructed to press the J key with the right forefinger. Otherwise, they were instructed to press the F key with the left forefinger. The 2-back task was similar to the 1-back task except that it required participants to compare the current letter with the second letter preceding it. In the TBPM task, participants were instructed to press the 1 key per minute. They could check the time by pressing the space key with a thumb at any time, and the time reminder would display for 1 s on the screen. All the materials were presented in 22-point font at the center of an 18^″^ LED monitor. The experimental procedure controlled by E-Prime 1.1 software program running on some DELL computers.

#### Procedure

The procedure was tested in a predetermined order. First, the instruction for the ongoing task was presented on the screen. Participants were told that they should make a decision by pressing the appropriate key as soon as possible. Under the easy task condition, they just needed to implement the 1-back task. And other participants under the difficult task condition performed the 2-back task. After understanding the task requirements, they were required to practice the ongoing tasks for 30 trials. Each trial started with a fixation (“+”) at the center of the screen for 300 ms. Then, a capital letter would appear for a maximum of 4000 ms and disappear if the corresponding response was made. Then, the trial ended. There was no delay between each two stimuli, so the intertrial interval was 0 ms. We set the probability of all letters appearing in the program to be the same, but the ratio of participants making same and different judgments was 1:2. After some practice trials, the instruction for the prospective memory task was presented. Participants were informed that the prospective memory task needed to be performed simultaneously with the ongoing task. They could check the time at any time and the time reminder would appear below the center of the screen. Next, participants began the formal experiment, which included the training stage and testing stage. In the training stage, participants implemented the 1-back task under the easy task condition and the 2-back task under the difficult task condition. The participants in the experimental condition were required to practice both the ongoing task and prospective memory task, but those in the control condition practiced only the ongoing task. The training stage started with the PM instructions and lasted for more than 30 min, during which participants were required to perform more than 1400 ongoing tasks and 30 prospective memory tasks in total. Participants could receive feedback every time they completed a TBPM task and take a break every 10 TBPM tasks. The final phase was the testing stage, which included 4 prospective memory tasks and more than 180 ongoing tasks. The interval between the training stage and the testing stage was 1 h. At the end of the experiment, all the participants were asked whether they remembered the prospective memory task.

### Results

**FIGURE 1:**
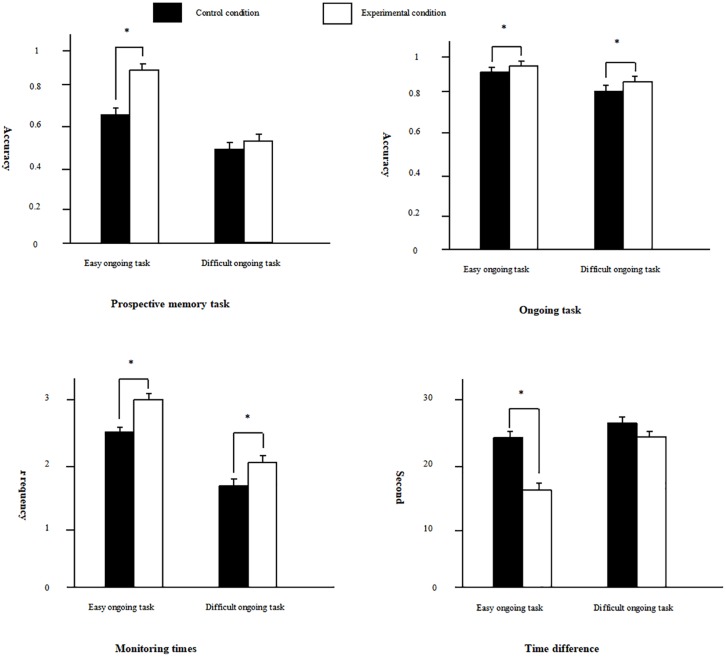
The results of Experiment 1, separately for prospective memory task performance, ongoing task performance, monitoring times, and time difference. Black bar represents control condition, white bar presents experimental condition, and the asterisk represents a significant difference between two conditions. In the figure, we only identify the differences between the control condition and the experimental condition on these indicators.

If the prospective memory task was executed within 5 s before and after the required time point of the TBPM task, the response was regarded as correct. Participants who forgot the prospective memory task and whose ongoing task scores were beyond three standard deviations were eliminated. The performances of ongoing task and prospective memory task in Experiment 1 were described in Table 1 and Figure 1. We first analyzed the prospective memory performance. A 2 × 2 ANOVA showed that the main effects of the training conditions and task difficulty were all significant, *F*(_1_, _125_) = 8.08, *p* < 0.01, η*_*p*_*^2^ = 0.06, *F*(_1_, _125_) = 23.66, *p* < 0.001, η*_*p*_*^2^ = 0.16. The interaction between training conditions and task difficulty was also significant, *F*(_1_, _125_) = 4.01, *p* < 0.05, η*_*p*_*^2^ = 0.03. The simple effect analysis revealed that the performances of prospective memory under the easy task condition were significantly higher in the easy task condition than those in the difficult task condition in both the control condition and the experimental condition, *F*(_1_, _125_) = 4.09, *p* < 0.05, η*_*p*_*^2^ = 0.03, *F*(_1_, _125_) = 23.45, *p* < 0.001, η*_*p*_*^2^ = 0.16. Under the easy task condition, the performance in the experimental condition was significantly better than that in the control condition, *F*(_1_, _125_) = 12.29, *p* < 0.001, η*_*p*_*^2^ = 0.09.

**TABLE 1:** The performances of ongoing task and prospective memory task in Experiment 1.

		**Ongoing task**		**Prospective memory task**		
			
**Training conditions**	**Task difficulty**	**ACC**	**RT**	**ACC**	**Monitoring times**	**Time difference**
Control	Easy task	0.91 (0.05)	636 (138)	0.64 (0.30)	2.50 (0.77)	24.07 (7.25)
	difficult task	0.81 (0.06)	857 (117)	0.50 (0.35)	1.88 (0.88)	26.24 (6.65)
Experimental	Easy task	0.93 (0.03)	575 (136)	0.89 (0.16)	3.03 (1.01)	16.30 (8.30)
	Difficult task	0.85 (0.05)	725 (118)	0.54 (0.31)	2.15 (0.63)	24.44 (7.62)

Next, the ongoing task performance was analyzed. We conducted a 2 × 2 ANOVA on the accuracy rate, and the results revealed that the main effects of the training conditions and task difficulty were all significant, *F*(_1_, _125_) = 11.63, *p* < 0.001, η*_*p*_*^2^ = 0.09, *F*(_1_, _125_) = 108.84, *p* < 0.001, η*_*p*_*^2^ = 0.46, and the scores of the experimental condition and the easy task condition were better than those of control condition and difficult task condition, respectively. A 2 × 2 ANOVA on the reaction time of the ongoing task showed that the main effects of the training conditions and task difficulty were also significant, *F*(_1_, _125_) = 18.00, *p* < 0.001, η*_*p*_*^2^ = 0.13, *F*(_1_, _125_) = 67.18, *p* < 0.001, η*_*p*_*^2^ = 0.35, the reactions were faster under the easy task condition and experimental condition.

We further analyzed the indicators of time monitoring. First, we conducted a 2 × 2 ANOVA on monitoring times, and the results revealed that the main effects of training conditions and task difficulty were significant, *F*(_1_, _125_) = 7.40, *p* < 0.01, η*_*p*_*^2^ = 0.06, *F*(_1_, _125_) = 25.85, *p* < 0.001, η*_*p*_*^2^ = 0.17, there were more monitoring times on experimental condition and easy task condition. A 2 × 2 ANOVA on the time difference revealed that the main effects of the training conditions and task difficulty were significant, *F*(_1_, _125_) = 13.18, *p* < 0.001, η*_*p*_*^2^ = 0.10, *F*(_1_, _125_) = 15.29, *p* < 0.001, η*_*p*_*^2^ = 0.11. The interaction between the training conditions and task difficulty was also significant, *F*(_1_, _125_) = 5.13, *p* < 0.05, η*_*p*_*^2^ = 0.04. The simple effect analysis revealed that under the difficult task condition, the time difference of the experimental condition were shorter than that of the control condition, *F*(_1_, _125_) = 18.08, *p* < 0.001, η*_*p*_*^2^ = 0.13. In addition, the time difference of the easy task were shorter than that of the difficult task under the experimental condition, *F*(_1_, _125_) = 18.89, *p* < 0.001, η*_*p*_*^2^ = 0.13.

### Discussion

The dual-task paradigm was adopted in the present study. Participants needed to perform the ongoing task and prospective memory task simultaneously, so that the two tasks would interact with each other. There were two goals for Experiment 1. The first goal was to explore whether the practice effect on TBPM existed under different task difficulty conditions. The results of worse performance of the ongoing task under the difficult task condition revealed the effective manipulation of the task difficulty. The prospective memory performance revealed that the easy task could benefit from practice, but the difficult task could not. The findings were consistent with the hypotheses of Experiment 1, suggesting that the practice effect on TBPM was affected by the difficulty of the ongoing task. However, we validated the results of previous study (Waldum et al., 2016) only under easy ongoing task condition.

The second aim of Experiment 1 was to explore the cognitive processing mechanism of the practice effect on TBPM. The results of the ongoing task accuracy rate and reaction time indicated that the ongoing task also benefited from the training for the prospective memory task, indirectly demonstrating that the attention resources required for the prospective memory task were reduced from the perspectives of the attention resource theory and the prospective interference effect. Besides, the monitoring times of the experimental condition were more than those of control condition, revealing that participants in the experimental condition paid more attention to temporal information. The results of the time difference showed that the effectiveness of time monitoring had been improved under the easy task condition. This finding was consistent with the prediction of the DAT and the hypothesis of the present study. However, the effectiveness of time monitoring did not improve under the difficult condition.

The above results revealed that the practice effect on TBPM existed in the easy background task condition, but not in the difficult background task condition. In addition, the effectiveness of time monitoring improved only in the easy task condition. Therefore, the practice effect was closely related to the effectiveness of time monitoring. However, under the experimental condition, participants also paid more attention to temporal information even in the difficult task. It was not consistent with the views of earlier studies (Vanneste et al., 2016) and the results in the easy task, which indicated that the practice effect was limited by the difficulty of ongoing tasks. The results above indicated that the key to the practice effect lies in the quality of attention rather than the quantity. Moreover, we speculated that prospective memory task required less attention resources after training because of the better performance of ongoing task. The reason might be that practice also improved the automation of TBPM in a manner similar to the effect of practice on EBPM. More concretely, the accessibility of prospective memory intentions related to memory might be improved, leading to higher spontaneousity in the retrieval of intentions (Penningroth and Scott, 2013). We also found the phenomenon under the difficult ongoing task, but the practice effect did not exist under this condition. It was likely that, in the present study, the retrospective component of TBPM was too simple to determine the practice effect.

Through the above analyses, we found that the practice effect existed only in the easy background task, and that it might benefit from the improvement of the effectiveness of time monitoring. However, the difficult background task prevented the generation of the practice effect. In the studies using the dual-task paradigm, the difficult ongoing task usually occupied more attention resources, which exerted a negative impact on prospective memory (Smith and Hunt, 2014; Kaschel et al., 2016). The effect of background task difficulty on the practice effect on TBPM should be related to the amount of attention resources allocated to prospective memory task. In the difficult ongoing task condition, prospective memory task did not receive sufficient attention resources even after training. It would affect the accuracy of time estimation (Taatgen et al., 2007; Coyne et al., 2009), which might reduce the effectiveness of time monitoring. If we further provided further attention resources to prospective memory tasks, for example, by providing time delay after each ongoing task stimulus response, it might be possible to produce the practice effect under the difficult task condition due to getting enough attention resources for prospective memory. We planned to verify this hypothesis in Experiment 2.

## Experiment 2

In Experiment 1, we did not find the practice effect in the difficult task. We speculated that the reason was that the prospective memory task was disturbed by insufficient attention. Therefore, we planned to add a delay after each difficult ongoing task to examine whether this change could produce the practice effect. The 2-back task was also adopted to create difficult background task. Because TBPM needs more self-initiated attention resources than EBPM (Voigt et al., 2011; Cruz et al., 2017), we set the delay to be as long as 600 ms, rather than the 200 ms specified by Loft and Remington. Finally, we further confirmed the cognitive mechanism of the practice effect again with the same indicators used in Experiment 1.

### Method

#### Participants and Design

Sixty-four university students participated in Experiment 2 for monetary compensation (30 RMB, about 4.5 dollars). They were tested individually. Their ages were between 18 and 23 years (*M* = 20.58, *SD* = 1.31). The experiment adopted single factor between-subject design. The independent variable was the training conditions, which included the control condition and the experimental condition. Participants were randomly assigned to the two conditions. According to the criteria mentioned in the results, we obtained thirty-four and thirty-one effective participants in the control condition and experimental condition, respectively. The research was approved by the Academic Committee of Southwest University.

#### Materials, Tasks, and Apparatus

We adopted the same materials, apparatuses, and prospective memory task as those used in Experiment 1. However, only the 2-back task was used for the ongoing task.

#### Procedure

The procedure was approximately the same as that used in Experiment 1 except for that we added a 600 ms blank delay after each ongoing task; thus the intertrial interval was 600 ms. The durations of the training stage and the testing stage in Experiment 2 were the same as those in Experiment 1. During the training stage, participants needed to practice more than 900 ongoing tasks and 30 prospective memory tasks. In addition, the testing stage consisted of more than 120 ongoing tasks and 4 prospective memory tasks.

### Results

**FIGURE 2:**
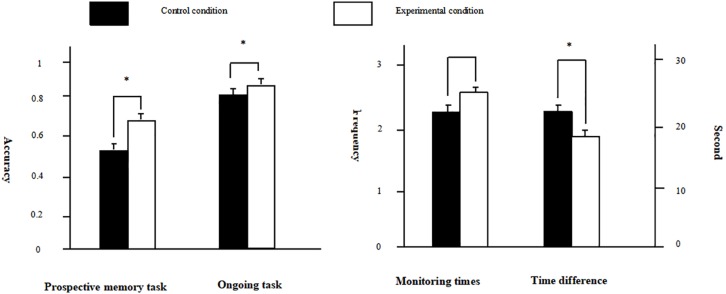
The results of Experiment 2, separately for prospective memory task performance, ongoing task performance, monitoring times, and time difference. Black bar represents control condition, white bar presents experimental condition, and the asterisk represents a significant difference between two conditions. In the figure, we only identify the differences between the control condition and the experimental condition on these indicators.

Participants who forgot the prospective memory task and whose ongoing task scores were beyond three standard deviations were eliminated. The performances of ongoing task and prospective memory task in Experiment 2 were described in Table 2 and Figure 2. First, the performance of prospective memory was analyzed. Participants were considered to have performed the prospective memory task correctly if they made the appropriate response within 5 s before and after the required time point of the TBPM task. A single-factorial ANOVA showed that performance in the experimental condition was better than that in the control condition, *F*(_1_, _63_) = 5.38, *p* < 0.05, η*_*p*_*^2^ = 0.08. We also compared the performance of the ongoing task, the results revealed that experimental condition had higher scores and faster response than control condition, *F*(_1_, _63_) = 5.65, *p* < 0.05, η*_*p*_*^2^ = 0.08, *F*(_1_, _63_) = 11.75, *p* < 0.001, η*_*p*_*^2^ = 0.16. The indicators of monitoring times and time difference were further analyzed. The results revealed that there was no difference between the two training conditions in time monitoring times, but the time difference under experimental condition was shorter than that under control condition, *F*(_1_, _63_) = 6.11, *p* < 0.05, η*_*p*_*^2^ = 0.09.

**TABLE 2:** The performance of ongoing task and prospective memory in Experiment 2.

	**Ongoing task**		**Prospective memory task**		
		
**Training conditions**	**ACC**	**RT**	**ACC**	**Monitoring times**	**Time difference**
Control	0.86 (0.06)	795 (104)	0.52 (0.30)	2.31 (0.87)	23.61 (6.55)
Experimental	0.89 (0.04)	701 (117)	0.67 (0.24)	2.58 (0.86)	19.64 (6.35)

Next, we further compared all the indicators of the two experiments in difficult task condition with a 2 × 2 ANOVA. For the TBPM performance, we found that the main effect of training conditions was marginal significant, *F*(_1_, _123_) = 3.40, *p* = 0.068, η*_*p*_*^2^ = 0.027, the accuracy of experimental condition was better than that of control condition. For the ongoing task performance, the results revealed that participants in the experimental condition had higher accuracy and faster response than those in the control condition, *F*(_1_, _123_) = 11.00, *p* < 0.001, η*_*p*_*^2^ = 0.08, *F*(_1_, _123_) = 30.94, *p* < 0.001, η*_*p*_*^2^ = 0.20. And participants in Experiment 2 had higher accuracy and faster response than those in Experiment 1, *F*(_1_, _123_) = 20.27, *p* < 0.001, η*_*p*_*^2^ = 0.14, *F*(_1_, _123_) = 4.59, *p* < 0.05, η*_*p*_*^2^ = 0.04. For the monitoring times, we found that participants in Experiment 2 had less monitoring times than those in Experiment 1, *F*(_1_, _123_) = 8.76, *p* < 0.01, η*_*p*_*^2^ = 0.07. For the time difference, the results revealed that the time difference in Experiment 2 was shorter than that in Experiment 1, *F*(_1_, _123_) = 9.50, *p* < 0.01, η*_*p*_*^2^ = 0.07, and the time difference in experimental condition was shorter than that in control condition, *F*(_1_, _123_) = 5.70, *p* < 0.05, η*_*p*_*^2^ = 0.04.

### Discussion

We had found that difficult and fast-paced ongoing task would interfere with the practice effect in Experiment 1. The main purpose of Experiment 2 was to explore the possibility that whether the practice effect existed under the difficult background task when we slowed down the pace of ongoing task through adding a 600 ms delay between every two ongoing tasks. By comparing the monitoring times of Experiment 1 and Experiment 2, we found that adding the delay could increase the attention to temporal information. The performance of the prospective memory task showed that practice improved the scores for the TBPM tasks after training, revealing that the delay reduced the limitation of practice effect in the difficult background task condition. We further validated the results of previous study (Waldum et al., 2016).

In addition, we planned to verify the cognitive mechanism of the practice effect. First, we found a significant effect on both the accuracy rate and the reaction time of the ongoing task. This finding revealed indirectly that training for the prospective memory task reduced the dependence of prospective memory on attention resources. However, we did not find a difference between the control condition and experimental condition in monitoring times. It was also consistent with the easy task condition of Experiment 1 and suggested that there was not necessarily a relationship between the practice effect and the investment of attention for the temporal information processing. The indicator of time difference showed that time monitoring was more effective in the experimental condition, which was consistent with the predictions of the delay theory and the present study. In the difficult task of Experiment 1, we did not find both the practice effect and the improvement of effectiveness, but we found both phenomena in Experiment 2. It suggested that the practice effect was closely related to the effectiveness of time monitoring. In addition, when the delay was added, we found better ongoing task performance. It suggested that the ongoing task could also benefit from the delay. Besides, compared with the difficult task condition in Experiment 1, we also found better prospective memory performance and smaller time difference in Experiment 2. It further verified that there was a close relationship between the practice effect on TBPM and the time difference.

In general, the practice effect was found when we added a delay. Meanwhile, participants reduced the cost to TBPM, but their attention to temporal information did not declined. In addition, as predicted by the DAT, the effectiveness of time monitoring improved after training.

## General Discussion

This study addressed questions about the practice effect on TBPM. The first goal was to explore whether the practice effect existed. This problem was tested in Experiment 1, in which we adopted the 1-back task and 2-back task to create an easy background condition and a difficult background condition, respectively. The results revealed that the practice effect was found only in the easy ongoing task condition, but there was no obvious improvement of prospective memory performance after practice under the difficult ongoing task condition. This finding was consistent with our research hypothesis and a previous study (Waldum et al., 2016). It indicated that the practice effect was affected by the difficulty of the ongoing task. The reason that practice did not work under the difficult background task condition was probably related to the insufficient attention input for the TBPM task. Attention itself was modulated by cognitive load. High working memory load would result in a reduced capability to filter out task-irrelevant information (Lavie and De, 2005; Santangelo and Macaluso, 2013). Both ongoing tasks and prospective memory tasks competed for limited attention resources (Einstein et al., 2005; Pedale et al., 2017). When the background task was difficult, most of the participants’ attention was occupied by the ongoing task, resulting in insufficient processing of the TBPM task. These views were confirmed by the phenomenon of monitoring times decreasing under the high-difficulty ongoing task condition in Experiment 1. However, different components of the TBPM task might show different influences of the difficult ongoing task. The retrospective component mainly contained retrospective memory. Less difficult retrospective memory can be trained to reach the state of spontaneous processing after a great deal of practices (Jennings and Jacoby, 2003; Gardner et al., 2013) and makes it less sensitive to difficult background task. The memory content used in the present study was relatively simple, so the retrospective component should be less affected by the background task difficulty. Besides, the prospective component mainly included temporal information processing, such as time estimation. And the ability of time estimation was not easy to improve through short-term training when filling tasks were difficult (Taatgen et al., 2007). In addition, TBPM requires self-initiated attention resources, and the temporal information processing in prospective component could not attain a degree of spontaneous processing, which made TBPM easily restricted by the cognitive load (Dong and Huang, 2010; Voigt et al., 2011). Therefore, the effect of task difficulty on the practice effect was most likely related to the prospective component.

Whether the practice effect existed under the difficult ongoing task condition when a delay was added? We speculated that participants devoted less attention to TBPM task when the background task was difficult, which interfered with the production of the practice effect. In Experiment 1, we found less monitoring times under the difficult ongoing task condition, validating the speculation. The delay theory suggested that participants could get more attention resources if the time was prolonged (Heathcote et al., 2015). Therefore, we added a 600 ms delay after each ongoing task. The indicator of monitoring times was significantly improved in Experiment 2, proving that the addition of the delay played an important role in increasing participants’ attentional input to the TBPM task. As expected, we found the practice effect in Experiment 2, though the difficulty of the ongoing task did not directly decrease compared with the difficult ongoing task in Experiment 1. Besides, we also found ongoing tasks were also benefit from the delay. It might be that TBPM tasks took advantage of the attention resources provided by the delay, thereby reducing the interference with ongoing tasks. Based on the above findings, we infer that attention can simultaneous influence the practice effect and the effectiveness of time monitoring.

The second purpose was to test the cognitive mechanism of the practice effect. We guessed that TBPM might benefit from practice in three aspects. The first aspect was the improvement of the effectiveness of time monitoring, which was the prediction of DAT. We found that there was a positive relationship between the time difference and the practice effect, as the reduction of the time difference and the practice effect always appeared simultaneously. According to DAT, repeated training for TBPM tasks could improve the effectiveness of attention, which might related to the improvement of time estimation ability. Therefore, the change in the time difference was likely to be related to the ability of time estimation. During the 30 TBPM task exercises, participants performed the same number of time estimation training attempts. The ability of time estimation became more precise after many training attempts, and the difference between subjective time and objective time was became shorter (Bliss et al., 2012; Panagiotidi and Samartzi, 2013; Healy et al., 2015). When executing the TBPM tasks, participants usually relied on the ability of time estimation to monitor time to ensure that they could perform the prospective memory task accurately. Therefore, we inferred that the essence of the improvement of monitoring effectiveness was the enhancement of time estimation capability. The second aspect was the increase in overall attention input. We found more monitoring times of the experimental condition in Experiment 1, no matter how difficult the ongoing task was. However, the practice effect was found only in the easy ongoing task condition. Therefore, there was no inevitable relationship between monitoring times and the practice effect. In Experiment 2, we did find no difference in monitoring times between the experimental condition and the control condition. Increasing the delay eliminated the discrepancy in attentional input between the different training conditions. The reason might be that the delay provided sufficient time for participants to process temporal information under different levels of ongoing task difficulty. The comparison of monitoring times between Experiment 1 and Experiment 2 also confirmed that participants’ attention to temporal information significantly increased when the delay was added, which verified our hypothesis. Besides, we also found that the time difference became shorter when the delay was added, which revealed that delay increased the effectiveness of attention. It also indirectly proved that there was a close relationship between the practice effect and the effectiveness of attention. In sum, we still found no positive connection between attention input and practice effect. The third aspect was that practice might improve the spontaneous processing level of retrospective memory in the retrospective component. EBPM did not contain temporal information processing, so it had the possibility of spontaneous processing through practice (Einstein et al., 2005). Many studies also found evidence of spontaneous processing after the exercises of EBPM tasks (Rose et al., 2015; Guo et al., 2016). In addition, the retrospective components between EBPM and TBPM were the same. Therefore, the dependence of TBPM’s retrospective component on attention might also be reduced by practice. We found that the scores of the ongoing tasks improved under experimental condition in both Experiment 1 and Experiment 2, which indirectly proved that practice decreased TBPM’s dependence on attention resources.

It was remarkable that, based on the above analysis of the processing mechanism of the practice effect, training to TBPM task changed the input and distribution of attention. On the one hand, practice promoted the spontaneous processing of retrospective memory in the TBPM task, decreasing the cost of attention for the TBPM task. On the other hand, participants allocated the saving resources to the ongoing task and temporal information. But the key to the practice effect lies in the improvement of the effectiveness of time monitoring. Besides, to some extent, time monitoring was the external form of time estimation. Participants first estimated the clue of the TBPM task, and then spontaneously checked the time to get feedback. If the participants’ time estimation ability was improved, it would be shown by an improvement in the effectiveness of time monitoring. Therefore, the essence of improving the effectiveness of time monitoring was probably the enhancement of individual time estimation ability.

To summarize, we systematically explored the practice effect on TBPM. The 1-back task and 2-back task were adopted to create easy and difficult background tasks, respectively, in Experiment 1. We found the practice effect only under the easy ongoing task condition. In Experiment 2, the pace of the difficult ongoing task was slowed and the practice effect was found. In addition, we verified three possible reasons for the practice effect. The results revealed that the improvement of the effectiveness of time monitoring was closely related to the emergence of the practice effect.

There were several limitations to this study. First, the effect sizes of the reported effects were relatively small in the present study. The significant effects might be due to collecting a good number of participants in each group. Besides, we concluded that the practice effect was closely related to the effectiveness of time monitoring. However, there were many other factors that could contribute of the observed effect, such as anxiety. Participants’ emotional state was not assessed in the present study, so we could not rule out emotional impact. In addition, the time interval of 1 min was adopted in the present study. But it was a manageable time interval. There should be difference between the long time interval and the short one in cognitive mechanism, which might also affect the practice effect. In addition, we found that the practice effect was affected by the difficulty of the background task in Experiment 1. But it was drawn from short-term training in the TBPM task. Some studies showed that the cognitive resources required for retrospective memory, including working memory, significantly decreased after a long period of training (Morrison and Chein, 2011; Melby-Lervåg and Hulme, 2013). In addition, long-term perception of specific stimuli improves the accuracy of the time interval estimation (Szelag and Skolimowska, 2012; Schultz et al., 2013). Therefore, long-term training for TBPM task might make the practice effect eliminate the restrictions of the background task difficulty because of the steady improvement of the related capabilities. Besides, the indicators adopted in the present study also had limitations. We used monitoring times and time difference to represent attention input and attention effectiveness in temporal information processing, but temporal information processing contained many other aspects, for example, time estimation. However, no direct measurement was available in the present study.

## Ethics Statement

All participants provided written-informed consent to the study protocol that was approved by the Ethics Committee of the Southwest University and carried out in accordance with the Declaration of Helsinki.

## Author Contributions

YG performed the analysis. YG and PL performed the study and wrote the final report. XH was responsible for the design and planning of the study.

## Conflict of Interest Statement

The authors declare that the research was conducted in the absence of any commercial or financial relationships that could be construed as a potential conflict of interest.
